# EPIPAGE 2: a preterm birth cohort in France in 2011

**DOI:** 10.1186/1471-2431-14-97

**Published:** 2014-04-09

**Authors:** Pierre-Yves Ancel, François Goffinet

**Affiliations:** 1 INSERM, U_1153, Epidemiology and Biostatistics Sorbonne Paris Cité Center, Obstetrical, Perinatal and Pediatric Epidemiology Team, Maternité Port-Royal, 53 avenue de l’Observatoire, Paris 75014, France; 2 Paris Descartes University France, Paris, France; 3 URC - CIC P1419, Cochin Hotel-Dieu Hospital, Assistance Publique – Hôpitaux de Paris, Paris F-75014, France; 4 Maternité Port-Royal, DHU Risk in Pregnancy, Cochin Hotel-Dieu Hospital, Assistance Publique – Hôpitaux de Paris, Paris F-75014, France

**Keywords:** Preterm births, Cohort, Population-based study

## Abstract

**Background:**

Children born at low gestational ages face a range of risks and number of neonates surviving very preterm birth is increasing. We present the objectives and methods of a French national cohort of very and moderately preterm children, the EPIPAGE 2 study. It aims to examine short- and long-term outcomes of very preterm children and their determinants.

**Methods/Design:**

Eligible participants for this prospective population-based study include all infants live born or stillborn and all terminations of pregnancy between 22 and 31 completed weeks of gestation in all the maternity units in 25 French regions. In addition, a sample of moderate preterm births, i.e. births and late terminations at 32–34 weeks, was included in the same regions. In all, 7804 babies (stillbirths and live births) and terminations of pregnancy out of 8400 eligible births in France in 2011 that were either very (22–31 weeks) or moderately preterm (32–34 weeks) were included. Data on pregnancy, delivery, and neonatal events were extracted from the obstetric and neonatal records. The follow-up will collect information at corrected ages of one and 2 years and at 5, 8, and 12 years of age. Of the 4467 children discharged alive from the hospital and eligible for follow-up, 155 (4%) families refused further follow-up and 22 died before one-year of age. Finally, 4290 were included in the follow-up.

Eight additional projects investigating specific hypotheses among subsamples of the cohort by collecting specific data in addition to the core cohort data are being conducted to investigate 1) diagnosis of histologic chorioamnionitis, 2) early biomarkers of child health, 3) attitudes of care for extremely preterm infants, 4) painful procedures in neonatal intensive care units, 5) neonatal MRI cerebral abnormalities and their relation to executive functions, 6) associations between early gut colonization and early and late onset diseases, 7) impact of neonatal nutrition on child development, and 8) mother-infant attachment.

**Discussion:**

This project seeks to provide new data on the prognosis and etiology of very preterm birth and to assess related medical practices. Accordingly, it should lead to the development of new strategies of management and prevention in high-risk babies.

## Background

About 10 to 12% of births in the United States of America and 5 to 7% in European countries occur before the pregnancy reaches term [1,2]. In France, each year, almost 35 000 babies are born between 35 and 36 weeks (4–4.5%), 13 000 between 32 and 34 weeks (1.5%), and 13 000 (1.5%) at less than 32 weeks, i.e., very preterm [2]. Preterm birth, especially very preterm birth, is a major cause of neonatal mortality, morbidity, and disability. Because changes in medical practices and organization of care continue to have a strong influence on the outcome of these very preterm births, data about this population require regular updating. In the absence of routine data collection in France, population-based cohort studies are the methodology of reference for assessing the prognosis of high-risk children and determinants of long-term adverse outcomes.

The EPIPAGE 1 study included all very preterm babies born at 22–32 weeks in 9 French regions in 1997. It showed that rates of hospital mortality (15%), cerebral lesions (26% cerebral hemorrhage and 21% white matter damage), and disability (almost 40% of very preterm survivors had mild to severe disability at 5 years of age) remained high [3,4]. In recent years, European population-based studies have been conducted in the United Kingdom (EPICure, 1995, Trent Region, 1999–2005) [5,6], Belgium (EPIBEL, 1999–2000) [7], and Norway (1999–2000) [8]. These studies, which have primarily focused on populations of extremely premature children and those at the threshold of viability (less than 26 weeks gestation) born between 1995 and 2000, showed that more than 50% died before discharge and almost 50% of survivors developed moderate to severe disability [5,8].

The context of preterm births has changed over the last decade. In France, the comparison of data from EPIPAGE in 1997 with those of the MOSAIC project in 2003 for very preterm infants born between 24 and 31 weeks in the Paris area showed that the number of live births increased by 12% and that of survivors by 20% [9]. Two population-based studies investigating trends from the late 1990s through the early 2000s in health outcomes of extremely preterm babies born in Australia and the United Kingdom reported no change in survival among babies born at 22–23 weeks [6,10]. They differed, however, in their results for babies born at 24–25 weeks. In the Trent region, UK, Field [6] reported an increase in survival from 20% to 36% at 24 weeks and from 47% to 59% at 25 weeks, whereas survival rates did not change in the Australian state of Victoria [10]. The US NICHD Neonatal Research Network reported stable mortality between 2003 and 2007 among babies born before 28 weeks [11]. Although differences between countries in attitudes of care for babies born extremely preterm may explain these differences, too little is known about the underlying decision-making process. This aspect needs to be investigated.

Studies of time trends for cerebral palsy and neurodevelopmental disabilities among very preterm children have yielded conflicting results. Data from European registries showed that the rates of cerebral palsy and visual deficiencies fell from the 1980s to the mid-1990s among very preterm babies [12-15], whereas cohort studies reported either no change or an increase [16]. Although cognitive impairment remains one of the most serious problems among very and extremely preterm children, no clear time trend has been identified [17].

Changes have also occurred in the organization of care and in medical practices. In France, the National Perinatal Program (2005–2007) has set up perinatal networks to organize and coordinate the support and care of women at high risk for preterm delivery, but few data evaluating these networks are thus far available. Changes in neonatal care include the use of new protocols, notably for neonatal nutritional and developmental care [18]. Many pre- and postnatal drugs (tocolytics, low-dose aspirin, sedation) have entered general use without assessment of their long-term effects. Structured follow-up to ensure continuing care for very preterm babies after their discharge from the hospital is more frequent nowadays. There is, however, no agreement on specific protocols, and the question of effects on longer term outcomes remains open, in view of the weak level of evidence for the efficacy of interventions. Finally, many questions related to the causes and consequences of very preterm birth, including family burden, must still be explored.

These changes in the prognosis and management of very preterm infants raise multiple questions: what are the short- and long-term consequences of very preterm birth today? Are recent changes in medical practices and organization of care influencing child health and development? What are the mechanisms of long-term adverse outcomes? Do improvements in the identification of clinical, biological, and imaging factors help to better predict children’s motor, cognitive, behavioral, and health prognosis?

Accordingly, new population-based cohort studies have recently been launched. These include EPICure 2 in the United Kingdom [19], 12 years after the first EPICure study, and the EXPRESS study in Sweden [20], both covering infants born before 27 weeks. Given the diversity of medical practices at the limits of viability, extremely preterm birth should also be studied in other countries. It appears important to us as well to study births after 26–27 weeks because they are more numerous and account for the majority of children with developmental deficits. This paper describes the objectives and methods of the EPIPAGE 2 cohort study, a large population-based prospective study launched in 2011 in France.

### Objectives

The EPIPAGE 2 study aims to: 1) describe short- and long-term outcomes in very and moderately preterm babies and their families; 2) study changes in medical practices and organization of care and assess their impact on child health and development; and 3) explore the etiology of preterm birth and identify early predictors of health and developmental problems.

## Methods/Design

### Study design and population

EPIPAGE 2 is a population-based prospective study scheduled to follow children up to the age of 12 years. Eligible participants include all infants live born or stillborn and all terminations of pregnancy between 22 and 31 completed weeks of gestation in all the maternity units in 25 French regions (21 of the 22 metropolitan regions and 4 overseas regions) during the inclusion period. The only region that did not participate accounted for 18 415 births in 2011, i.e., 2.2% of all births in France. In addition, a sample of moderate preterm births, i.e., births and late terminations at 32–34 weeks, was included in the same regions. The study began on March 28, 2011, and ended on December 31, 2011. The population eligible for follow-up includes all children alive at discharge from neonatal intensive care or special units or maternity wards whose parents have not declined to participate. This study will also use a sample of children born at term for control purposes. This group will be extracted from the population of the Elfe study, a contemporary cohort with a 20-year planned follow-up of 18 500 children born at or near term in 2011, in 344 randomly selected public and private maternity units in metropolitan France ( http://www.elfe-france.fr). Information on the Elfe study is described elsewhere [21].

### Sample size/power calculation

The sample size was calculated to obtain a number of children at each week of gestation sufficient to 1) assess the frequency of adverse events with reasonable precision, i.e., the estimated value must lie within 20% of the true value (relative accuracy of 20%), 2) demonstrate differences in prognosis between preterm and term infants but also between preterm groups, with a statistical power of 80%, and 3) develop specific projects for subpopulations of very preterm babies. Table 1 reports the numbers of children required to be included at birth (live births only) and in the follow-up. Estimations were based on the frequency of the main health and developmental disorders expected in each gestational age range [4-7]. Table 1 also reports the number of children included in the study by gestational age groups.

**Table 1:** Required numbers of children to be included and numbers of children included

	**22-26 weeks**	**27-28 weeks**	**29-30 weeks**	**31 weeks**	**Total 22–31 weeks**	**32-34 weeks**
Expected % of all births (1)	0.15-0.20	0.20	0.25-0.30	0.40		2.0
Expected survival rates % (2)	50	80-85	90-95	95		98-99
Expected severe deficits among survivors % (3)	20	15	5	3-4		1-2
Number of children required for follow-up	450	750	900	800	2900	1200
Number of live births to be included	900	900	1000	850	3650	1200
Number of live births registered in EPIPAGE 2	1130	899	1265	932	4226	1340
Number of live births included in EPIPAGE 2 (parental agreement)	1054	857	1190	862	3963	1206
Number of survivors included in the follow-up	552	740	1146	836	3274	1193

(1)Estimated prevalence of births by gestational age categories.

(2)Estimated survival rates by gestational age categories.

(3)Estimated rates of severe deficits by gestational age categories.

### Recruitment

Recruitment took place at birth in all maternity units of the 25 participating French regions, after the parents received information about the study and agreed to participate. The required number of infants was provided by an 8-month recruitment period for extremely preterm births (22–26 weeks), from March 28 to November 27, 2011, in 7 regions (Alsace, Bourgogne, Guyane, Languedoc-Roussillon, Limousin, Midi-Pyrénées, and Rhône-Alpes) and from May 2 to December 31, 2011, in the remaining 18 regions (Aquitaine, Auvergne, Basse-Normandie, Bretagne, Centre, Champagne-Ardenne, Franche-Comté, Haute-Normandie, Ile-de-France, Lorraine, Nord-Pas-de-Calais, PACA-Corse, Pays de la Loire, Picardie, Martinique, La Réunion, and Guadeloupe), and by a 6-month recruitment period for very preterm births (27–31 weeks), from March 28 to September 25, 2011, in 7 regions and from May 2 to October 30, 2011, in the remaining 18. In addition, all children born at 32–34 weeks were included for 5 weeks from May 2 to June 5, 2011.

During the study period, 8400 births (stillbirths and live births) and terminations of pregnancy (TOP) were registered in the EPIPAGE 2 cohort study: 3261 were born extremely preterm (1081 (33.1%) TOP, 1050 (32.2%) stillbirths and 1130 (34.7%) live births), 3744 very preterm (292 (7.8%) TOP, 356 (9.5%) stillbirths and 3096 (82.7%) live births), and 1395 moderately preterm (19 (1.4%) TOP, 36 (2.6%) stillbirths and 1340 (96.0%) live births). Seven percent of the families refused to be included in the study at the stage of data extraction/collection from hospital records. However, status at birth, mortality, and a few perinatal data (gestational age, birth weight, and socioeconomic status) were available for them. Of the 4467 children discharged alive from intensive care and eligible for follow-up, 4% of the families refused and 22 died before one-year of age. Finally, 4290 were included in the follow-up.

In each region, a coordinating committee was set up specifically for the implementation of the study at the regional level. Contacts were identified in each maternity ward and each neonatal unit to supervise inclusions and data collection. During the recruitment, members of the regional coordinating committee visited all maternity units to ensure the identification of all eligible children.

### Data collection at birth

At birth and during the neonatal period, data were collected in the maternity and neonatal units. They were extracted from medical records and completed by questions to obstetrical and neonatal teams. The initial data collected about the pregnancy and delivery are intended to allow us to investigate specific pregnancy complications, including infectious, inflammatory, and vascular-placental diseases, medication use up to the moment of birth (tocolytics, magnesium sulfate, antenatal corticosteroids, and anesthesia), and decisions about termination of pregnancy, delivery, or limitations of care. The neonatal component was designed to capture the child's condition at birth, neonatal diseases, organization of care and management after birth, treatment and medication (sedation, postnatal corticosteroids, etc.), and abnormalities in early brain development and maturation, assessed by imaging techniques. We collected data on attitudes of care for extremely preterm births by describing situations in which intensive care was limited and palliative care provided at birth and situations where intensive treatment was initiated, pursued, and subsequently limited. Mothers were interviewed in the neonatal unit to obtain information about the family's social position and the mother's perception of her prenatal management. Each mother also completed a self-administered questionnaire assessing her mental health and the management of her baby in neonatal units, just before the baby’s discharge.

### Data management

Data extracted from maternity and neonatal records were completed directly online, with a secure interface for maintaining the confidentiality and privacy of data and personal information. Completed maternal interviews and self-administered questionnaires were sent for entry in the same dedicated database. Data entry and validation were performed as a continuous process. Programs were run to check missing data and range of responses.

### Data collection about obstetric and neonatal units

Another part of the EPIPAGE 2 study focused on the maternity and neonatal units that care for very preterm children. We distributed questionnaires to the medical teams of these units to collect data on their structural characteristics, organization, and policies and practices related to medical interventions and decision-making processes. This data collection took place in 2012 in 402 maternity units where very preterm deliveries occurred in 2011 and in 272 neonatal units. Ninety-one percent of maternity units and 100% of neonatal units completed the questionnaire.

### Follow-up

We plan to collect follow-up information at corrected age (CA) of 1 and 2 years and at 5, 8, and 12 years of age. The follow-up at 1 year corrected age has just ended. It comprised a questionnaire sent to the parents collecting information on postneonatal care (hospital admissions, medical visits, etc.), growth, respiratory diseases, including bronchiolitis, and treatments, maternal health, and the family’s social condition. At 2 year CA, questionnaires are being sent to the parents and to the physician providing care for the child. The questionnaire to the parents investigates child development with standardized scales, i.e., the Stages and Ages Questionnaire, the Modified-Checklist for Autism in Toddlers, and the short form of the MacArthur Communicative Development Inventory. It also collects data on child nutrition and maternal health. Physicians are asked to complete a short questionnaire on motor and sensory deficits. In regions where formal follow-up networks have been set up for the care of very preterm children, the 2-year (CA) medical data will be provided by the network's reference physicians.

Assessments of health status and development will be performed in regional examination centers at 5, 8, and 12 years of age. These check-ups will include a complete clinical examination and psychological tests and will evaluate, using standardized tools:

– Growth, assessed by standardized anthropometric measurements;

– Motor disorders, especially cerebral palsy and minor neuromotor dysfunctions. Cerebral palsy will be identified according to the diagnostic criteria proposed by the Surveillance of Cerebral Palsy in Europe (SCPE) network [22]. The Gross Motor Classification System [23] and the Manual Ability Classification System (MACS) [24] will be used to assess functional abilities. A standardized neurological examination will be used to explore fine motor disorders.

– Cognitive functions, assessed by the intelligence quotient (IQ), according to the principal validated scales, i.e., the Wechsler Intelligence Scale for Children (WISC-III or IV), Wechsler Preschool and Primary Scales Intelligence (WPPSI) [25], or the Kaufman Assessment Battery for Children Test [26]. Because these tools do not detect subtle disorders in visuomotor, language, memory, or executive functions, specific investigations will be conducted for these functions.

– Behavioral aspects of development, assessed by the Strengths and Difficulties Questionnaire [27], which is the most widely used and validated behavioral scale in France. This scale will be completed by an assessment of children’s symptoms of anxiety and depression.

– Quality of life (QoL) with a self-administered questionnaire, KIDSCREEN [28], and with the Child Health Questionnaire (CHQ), a generic instrument for measuring objective QoL and health status for children, both designed for the general population and children with various diseases.

At the ages of 5, 8, and 12 years, we will also collect data about educational achievement, school difficulties, special support at school, and special care outside school. The results of the national tests given at 12 years will also be collected.

Postneonatal care has evolved, and programs exist that aim to improve very preterm children’s prognosis. The implementation of these new practices need to be assessed in detail, however, to measure all their effects, beneficial or not, especially over the long term. There are various proposed interventions, which remain poorly described. Their description is the first step planned, and their evaluation, the second, because the level of evidence of interventions in this area is low [29].

### Data analysis

All statistical analyses must be pre-specified and approved by the steering committee. Participation refusals and children lost to follow-up will be compared with participating subjects for recorded perinatal factors such as gestational age, status at birth, neonatal care level, and region. Analyses will take place after the completion of each phase (neonatal period, ages 1 (CA), 2 (CA), 5, 8, and 12 years). A number of analyses will utilize the strength of the longitudinal design and multilevel multivariate statistical modeling methods to determine not only the neonatal predictors of outcomes, but also complications, care, and social environment over time.

### Ethics

Recruitment and data collection occurred only after families had received information and agreed to participate in the study. The data collected at birth and during the follow-up phases were and will continue to be totally anonymized. The only nominative file is the one containing the family addresses and contact information — the data that make the follow-up possible, but which are completely separated from all survey data. As required by French law and regulations, this study was approved by the national data protection authority (Commission Nationale de l’Informatique et des Libertés, CNIL n°911009) and by the appropriate ethics committees, i.e., the advisory committee on the treatment of personal health data for research purposes (CCTIRS: Comité Consultatif sur le Traitement de l'Information en matière de Recherche, approval granted November 18, 2010; reference number 10.626) and the committee for the protection of people participating in biomedical research (CPP: Comité de Protection des Personnes, approval granted March 18, 2011, reference CPP SC-2873).

### Project governance

The governance is consistent with the charter set forth by the French Institute for Public Health Research, which outlines the rights and responsibilities of research teams working together with very large cohorts for health research ( http://www.iresp.net). The project is coordinated at two levels: 1) at the national level by a Steering Committee responsible for the scientific project and organization of the study; this committee includes epidemiologists, pediatricians, obstetricians, and members of perinatal networks, from all participating regions of France; and 2) at the regional level, with a coordinating committee set up in each region specifically to implement the study there.

### Additional projects

EPIPAGE 2 has been designed to allow various research groups to develop additional projects aimed at investigating specific hypotheses among subsamples of the cohort by collecting specific data in addition to the core cohort data. Eight additional projects are already underway in the fields of placental histology (CHORHIST), genetics (BIOPAG), ethics, painful procedures in neonatal units (EPIPPAIN 2), cerebral imaging (EPIRMEX), nutrition (EPINUTRI and EPIFLORE), and mother-infant attachment (OLIMPE) (Table 2).

**Table 2:** Projects associated with EPIPAGE 2

**Projects (Investigator(s))**	**Objectives**
**CHORHIST** (G Kayem, D Subtil, Obstetricians)	To identify histological chorioamnionitis by examining the placentas of all children born between 22 and 31 weeks in 23 perinatal centers and to study it in relation to short- and long-term outcomes (N = 1406).
**BIOPAG** (P Boileau, Pediatrician, V. Benhammou, Biologist)	To determine genetic markers of mother and child and their relation to short- and long-term outcomes. Maternal and cord blood samples (DNA, RNA) were collected at birth in 14 perinatal centers (N = 149).
**ETHICS** (L Foix-L’Hélias, Pediatrician, Epidemiologist)	To assess situations in which limitation of care is discussed during prenatal or neonatal care and to analyze the decision-making processes that led to limitations, withdrawals, or substitutions of palliative care, or continuation of care among extremely preterm infants (live born, stillborn, and pregnancy terminations < 27 weeks) born in 18 perinatal centers (N = 419).
**EPIPPAIN 2** (R Carbajal, Pediatrician)	To analyze painful procedures in neonatal intensive care units and their relation to child health and development in all infants born very preterm in Paris-area level III facilities and included in the EPIPAGE 2 cohort (N = 562).
**EPIRMEX** (E Saliba, Pediatrician)	To study executive functions and language development during early childhood as a function of abnormalities detected by MRI at term among babies born at gestation ages of 26 to 31 weeks in 15 perinatal centers where conventional and advanced MRI techniques (3D MRI for the volume measurements, diffusion tensor images for tractography analysis) were available (N = 582).
**EPIFLORE** (MJ Butel, Microbiologist)	To study the development of the intestinal microbiota and to analyze the associations between these abnormalities and diseases of early childhood, childhood, and adolescence (e.g., allergies, metabolic syndrome, and diabetes) among very preterm children born in 19 perinatal centers (N = 728).
**EPINUTRI** (A Lapillonne, Pediatrician)	To study associations between neonatal nutrient intake (of polyunsaturated fatty acids, iron, and milk, with breastfeeding considered separately) and child development in a sample of very preterm infants, i.e., 22–31 weeks born in the Paris area (N = 325).
**OLIMPE** (JB Muller, Pediatrician, C Arnaud, Epidemiologist, C Lebeaux, Pediatrician, Epidemiologist)	To explore mother-infant attachment at hospital discharge and at 6 months among very preterm children born in 12 perinatal centers to 1) identify factors linked to the child's health status and to the organization of the neonatal unit that might influence interactions between mothers and their children; and 2) investigate whether the quality of early mother-infant interactions is associated with further child development and behaviour (N = 167).

### Collaborative projects

The EPIPAGE 2 cohort study is associated with a European project, EPICE (Effective Perinatal Intensive Care in Europe, http://www.epiceproject.eu), that is studying very preterm children born in 19 European regions in 2011–12, including three regions in France (Burgundy, Ile-de-France, and Nord-Pas de Calais). Its aim is to improve our understanding of how treatments or interventions that have been demonstrated to be effective are or not used in medical practice and what obstacles impede their widespread use. The data needed for the EPICE study for the three French regions is being collected as part of EPIPAGE 2. EPIPAGE 2 is also associated with the ELFE study ( http://www.elfe-france.fr), a contemporary cohort of babies born at 33 weeks or more. EPIPAGE 2 and ELFE are the two components of the RECONAI platform, a French research platform for birth cohorts. Information about very and moderately preterm births is being collected through EPIPAGE 2, and data about late preterm and term births through Elfe. EPIPAGE 2 and ELFE together will make it possible to study the events that influence development, health, and socialization of children from the fetal period through adolescence. The results will guide obstetric and pediatric care and public policy-making in the domains of family and childhood.

### Discussion

Very preterm children and their families face a range of medical, psychological, and social problems that raise specific questions at birth and thereafter. The need for information on the prevalence, causes, and consequences of preterm birth requires large population-based cohort studies with a long-term follow-up, especially in France, where the most recent detailed data about very preterm births date back to the EPIPAGE 1 study, 15 years ago. Especially with its associated projects, the EPIPAGE 2 study, which brings together a cohort of 4500 preterm children, offers an original approach. The establishment and follow-up of this cohort and the collection of such diverse data (social, clinical, biological, and imaging) will increase our understanding of how to 1) improve the care and management of preterm children, 2) identify predictors of long-term outcomes in various components of health in the light of recent progress in medical practices, particularly in the field of brain imaging, and 3) enhance both the organization of care and decisions about the continuation of treatment. Our findings should provide evidence to design new strategies of management and prevention and to inform trials of interventions evaluating their impact. It will also enable research teams to access this information and to test new hypotheses in this at-risk population.

## Abbreviations

CA: Corrected age; TOP: Termination of pregnancy.

## Competing interests

No potential conflict of interest relevant to this article was reported.

## Authors’ contributions

The protocol was developed by PYA, FG, and MK and approved by the scientific leaders in each region. All members of the EPIPAGE 2 Writing Group contributed to the design of the study, the development of the questionnaires in a series of workshops, and acquisition of the data. PYA drafted the manuscript. The final version has been approved by the entire EPIPAGE 2 writing group.

## Pre-publication history

The pre-publication history for this paper can be accessed here:

http://www.biomedcentral.com/1471-2431/14/97/prepub
